# Cervical Cancer Screening and Chinese Women: Insights from Focus Groups

**DOI:** 10.3389/fpsyg.2013.00048

**Published:** 2013-02-15

**Authors:** S. C. H. Chang, J. S. T. Woo, V. Yau, B. B. Gorzalka, L. A. Brotto

**Affiliations:** ^1^Department of Psychology, University of British ColumbiaVancouver, BC, Canada; ^2^Department of Obstetrics and Gynaecology, University of British ColumbiaVancouver, BC, Canada

**Keywords:** culture, pap testing, Chinese women, sexuality, Chinese medicine, cervical cancer, reproductive health, cancer screening

## Abstract

**Objective:** Despite extensive efforts to raise awareness, Papanicolaou (Pap) testing rates among Chinese women living in North America remain low compared with Euro-American women. Although the lower Pap testing rate and ensuing health repercussions among Chinese women are well characterized, mechanisms underlying such health disparities are not. The aim of this study was to use a qualitative approach to delineate such mechanisms. Qualitative approaches to understand constructs within the domain of sexual and reproductive health have been shown to be particularly appropriate, and offer a nuanced view of sexuality that is not afforded by traditional quantitative methods.

**Method:** We carried out two focus groups aimed at exploring how Mandarin-speaking and English-speaking Chinese women experience Pap testing (*N* = 12). The women were invited to partake in the focus groups from having participated in a large-scale quantitative study. Participants were all first-generation immigrants and their average age was 53-years-old. We used content analyses to analyze transcripts and extract themes.

**Results and Discussion:** The women heavily endorsed traditional Chinese medicine philosophy, conceptualizing physical health holistically, and valuing preventative measures over screening and interceptive measures. Pap testing was described as qualitatively different from other screening procedures, such that women assigned a sexually charged meaning to Pap testing, often discussing it in relation to sexual activity and promiscuity. Women expressed their preference for the compulsory and depersonalized manner that Pap tests are performed in their home country of China, as this lessens the embarrassment associated with undergoing Pap testing.

**Conclusion:** Three mechanisms may contribute to lower Pap testing among middle-aged first-generation Chinese immigrants: preference for Chinese medicine philosophy, perceived sexualization of Pap testing, and the institutionalization of medical care. Implications for improving the reproductive health of Chinese women are discussed.

## Introduction

Cervical cancer continues to be a significant health threat to women and a heavy burden on the health care system. Worldwide, cervical cancer is currently the sixth most deadly cancer (World Health Organization, 2012). An important safeguard against cervical cancer is the Papanicolaou (Pap) test, which involves the removal and biopsy of cells from the cervix. When conducted regularly, the Pap test not only enables early detection and treatment of cervical cancer, it can be preventative. From this procedure, premalignant and cancerous cells in the endocervical canal can be identified, which allows for the treatment of precancerous cells before they develop into cancer and thus preventing cervical cancer. In fact, since the introduction of widespread Pap testing, several countries have observed a steady decline in the incidence and mortality rate of cervical cancer (Canavan and Doshi, 2000).

Despite the benefits of the Pap test for cervical health and cancer prevention, it is significantly underutilized by Chinese women living in North America (Kagawa-Singer and Pourat, 2000; Hislop et al., 2004). Pap testing rates are consistently found to be lower among Chinese women than Caucasian women (Yu and Perrine, 1997; Kagawa-Singer and Pourat, 2000; Taylor et al., 2002; Tu et al., 2005), with devastating health consequences. Compared to the general population in North America, Chinese women have a higher incidence of cervical cancer and associated mortality (Archibald et al., 1993; Parkin et al., 1993), and present for treatment at more advanced stages (Jenkins and Kagawa-Singer, 1994).

The lower Pap testing rates among Chinese women persists even in the face of extensive health care changes. In 1955, the province of British Columbia in Canada instituted a program providing all women free access to the Pap test through their primary care providers. This initiative was deemed successful as cervical cancer morbidity and mortality dramatically declined by 85% and 75%, respectively since its launch (The Cervical Cancer Screening Program, 2005). Nonetheless, low Pap testing rates persisted among Chinese-Canadian women (Hislop et al., 2004). In 1994, the Asian Women’s Health Clinic was established as an additional attempt to elevate Pap testing rates among Chinese-Canadian women. This provided Chinese-Canadian women with a convenient source of reproductive health services and education from female Chinese health practitioners at no cost. Despite these efforts, Pap testing rates among Chinese women remained below the provincial average (Hislop et al., 2004; The Cervical Cancer Screening Program, 2007). Evidently, the availability of cancer screening resources alone was insufficient for overcoming this health disparity among Chinese women. In particular, although the prevalence of screening rates is well known, the precise mechanisms and motivations behind screening behaviors are poorly understood.

The current literature remains scant and mostly restricted to quantitative methodologies aimed at identifying predictors of Pap testing behavior in Chinese women (e.g., Yu et al., 2001; Chang et al., 2010). However, effective cancer control interventions targeting immigrant groups are best informed by a comprehensive understanding of their culturally based beliefs, knowledge, and practices (Olsen and Frank-Stromberg, 1993; Hubbell et al., 1995). That is, to mitigate the resistance to Pap testing among Chinese women, one must first understand the nature of the resistance within its cultural context.

### Chinese culture and immigration

Chinese culture is one of the world’s oldest and has been molded by thousands of years of sociopolitical, philosophical, and artistic movements. Chinese cultural values, norms, and attitudes are largely derived from Confucianism and Taoism. Some dominant Chinese values derived from these schools of thought include filial piety, propriety, humility, and emphasis on communal harmony over individual interests (Louie, 2008). Two aspects of Chinese culture most relevant to the phenomenon of underutilization of Pap testing among Chinese women may be those pertaining to sexuality and health/disease since Pap testing is a health examination of the female reproductive system.

With respect to sexuality, teachings from Confucianism and Taoism permeated Chinese thoughts and attitudes. Since the Song Dynasty (960–1276 AD) when Confucianism was declared the official state doctrine, Confucian classics came to be interpreted as sexually suppressive; sexual behavior was reserved for marriage and was viewed as serving a purely procreative role. This rigid interpretation has remained in favor for the last 1000 years and has likely formed the backbone for the present Chinese conservative attitudes toward sexuality (Ng and Lau, 1990). In contemporary culture, sex education in schools is minimal and parents as well as health professionals are reluctant to discuss sexuality and sexual information (Chan, 1986). Official and professional pronouncements in the People’s Republic of China often give an impression of strict sexual morals. For example, official educational material dispensed by the government frequently advised sexual expression be restricted within the bounds of marriage, and even then should be limited in frequency (Han, 1973; Yiu et al., 1985).

Instead of being another extension of Confucian and Taoist thought, Chinese beliefs toward health and disease originated from a series of influential works by great Chinese intellectuals. Approximately 2000 years ago, the semi-mythological Yellow Emperor, Huang Di systematically summarized and integrated years of Chinese medicine theory. The overarching belief in Chinese medicine is that the human body is a microcosm, a miniature universe. Just as nature included air, sea, and land, the body also is composed of *qi*, *moisture*, *blood*, *jing*, and *shen*, which are perceived as theoretical rather than physical entities. Disease states are likened to adverse climates within the body ecosystem and health is likened to a harmonious balance between *yin* and *yang* which produces a healthy state of *qi*. Neither health nor diseased states are believed to be static but are in constant flux and negotiation with one another. This runs counter to Western conception of disease to be a specific pathological process or structural abnormality that can be empirically and reliably detected (Beinfield and Korngold, 2003).

Currently, the Chinese in Canada constitutes the largest and fastest-growing visible minority (Statistics Canada, 2001) and in the United States, Chinese immigration has steadily increased since the 1930s (U.S. Department of Homeland Security, 2003), resulting in this group being the largest Asian ethnic subpopulation (U.S. Census Bureau, 2001). Having first arrived in the mid-1800s, the Chinese are also the oldest Asian community in North America. This first wave of Chinese immigrants were attracted by the booming North American economy and contributed significantly to the pivotal economic developments at the time, such as roadway building and gold mining (Hull, 1985). The last four decades have seen successive waves of Chinese immigration, often brought on by major historical events in Asia, such as the Tiananmen Square protest in 1989 and the return of Hong Kong to People’s Republic of China in 1997 (Mo, 1992; Lasky and Martz, 1993). These later surges of Chinese immigration have resulted in a substantial Chinese population living in North America that is predominantly foreign-born (Costa and Renaund, 1995; U.S. Census Bureau, 2001). While the early influx of Chinese immigrants generally acculturated to mainstream Western culture, the more recent arrivals, due to their lower degree of acculturation, poorer English fluency, and deeper cultural isolation, have become a group that requires special attention from public health professionals, but have thus far remain underserved (Gould-Martin and Ngin, 1981). Not surprisingly, Chinese individuals living in North America have been found to differ from their Caucasian counterparts with respect to attitudes and practices concerning sexuality and physical health in a direction that is consistent with their heritage culture. That is, Chinese individuals living in North America hold more conservative sexual attitudes and utilize more Chinese medicine services (Beinfield and Korngold, 2003; Ahrold and Meston, 2010; Meston and Ahrold, 2010).

While overall differences can be drawn between Chinese and Caucasian individuals living in North America, it is important to note that Chinese immigrants are not a homogeneous group. Hong Kong, Taiwan, and China diverge with respect to their cultural-social values, political ideologies, and economic infrastructures (Berndt et al., 1993; Cheung and Chow, 1999). Hong Kong was separated from the Chinese government and subjugated to British Rule in 1842. For the next century, Hong Kong functioned as a British colony and its current political, legal, and educational systems still reflect British philosophies. Alternatively, the economic and social systems in Taiwan have been heavily shaped by Japanese and American influences, stemming from the Sino-Japanese war in 1895 and American military support in the 1950s, respectively. With respect to its culture and religion, most people in Taiwan practice a unique mixture of Confucianism, Taoism, and Buddhism. In contrast to the political systems in Hong Kong and Taiwan, China has been a communist nation since 1949. One of the earliest acts of the Chinese Communist Party was to eradicate organized religion and Confucian values from Chinese culture (religious rights were later regained in 1978). The Party also injected Chinese culture with philosophies of Marxism–Leninism, with emphasis on efficiency and productivity. The people of China were also encouraged to look to the Party instead of their families for security and leadership (Cheung and Chow, 1999).

### Qualitative methods

Qualitative approaches are ideally suited for studying rich, complex, and poorly understood constructs and systems like culture (Schwandt, 1994). By examining variables in their multiplicity and context, qualitative approaches allow the researcher to gain a holistic and integrated overview of the context under study: its logic, arrangements, explicit, and implicit rules. Furthermore, qualitative methods are designed to describe, interpret, and understand human experiences, where the richness of individual experience is often preserved and highlighted, instead of omitted or reduced/simplified, as is often the case in quantitative methodologies (Miles and Huberman, 1994). In examining the lived experiences of people through their own words and stories, researchers can gain an “insider perspective” of a specific culture and a contextual understanding of cultural values and their relationships (Steckler et al., 1992; Gittelsohn et al., 1994; Sensky, 1996).

Due to these unique features, qualitative research is gaining momentum in health research. In fact, health educators are increasingly turning to qualitative methods to gain an understanding of minority health practices and concepts when developing health intervention and screening programs (Steckler et al., 1992). This type of research has already identified some important culture-specific obstacles to health care. For example, Mahloch et al. (1999) found that Cambodian refugees believe karma plays an important role in health outcomes and traditional practices can help prevent uterine disease in women. Also using a qualitative approach, Hubbell et al. (1995) developed a breast cancer control program for Latina women living in California and discovered that many Latinas did not endorse medically accepted risk factors (e.g., a family history of breast cancer), and instead believed that other factors (e.g., breast injury) increase cancer risk. Moreover, this study found that fatalistic attitudes (e.g., “breast cancer is a punishment from God”) were an obstacle to screening efforts among Latina women.

Considering the advantages of the qualitative paradigm, it is ideally suited for exploring the motivations and barriers associated with Pap testing rates in Chinese women. To our knowledge, there has been only one publication using a qualitative approach to understand Pap testing among Chinese women (Jackson et al., 2002). Using an iterative, anthropologic approach, where interviews were continually building on information discovered and validated during previous interviews so that each interview increased in detail, the authors identified only one culture-specific barrier to Pap testing among Chinese women; namely, the influence of Chinese medicine. The idea of Pap testing was found to conflict with the Chinese medicine emphasis on balance. Specifically, the idea of extracting cells as a form of screening and surgical removal of diseased tissue as a form of treatment are inconsistent with the traditional Chinese view that the cause of cancer is stagnant *qi* in the form of retained blood, “cold” and “wind,” and thus must be treated by restoring the balance of *qi*. As an extension to the principle of *qi*, many participants also believed that a person’s health is always in flux and that undergoing a Pap test in the absence of experienced symptoms may create unnecessary anxiety in a situation that would eventually self-correct anyway.

The goal of the present study was to build upon previous investigations of Pap testing behavior among Chinese women (Jackson et al., 2002; Woo et al., 2009). In adopting a qualitative approach, we capitalized on the ability to tap into interpersonal communication that emerges during group – a process not captured by traditional quantitative methods (Morgan and Kreuger, 1986). We believed that this method may also better recognize cultural values or group norms and identify shared and common knowledge. Another important advantage of focus groups is that it may encourage participation among those deterred by the formality and isolation of individual interviews (Kitzinger, 1995). The group setting can also promote participation in discussion of taboo topics. For these reasons, focus groups may capture voices who typically remain silent. Finally, the security of being among peers can also promote the group to express ideas they consider to deviate from the culture of the researcher (Morgan and Kreuger, 1986). Related to this, some researchers have noted that the focus group format generates more criticisms of mainstream institutions, compared to interviews (Geis et al., 1986; Watts and Ebbutt, 1987). Because of these unique advantages, focus groups are often used in cross-cultural research and studies examining why different sections of the population make differential use of health services (Kitzinger, 1995), rendering focus group an ideal method to investigate the usage of Pap tests among Chinese women. In the present study we adopted an open-ended focus group format to explore the experiences, motivations, and deterrents to Pap screening among two samples of Chinese women: one English and one Mandarin-speaking.

## Materials and Methods

### Participants

Thirteen women who self-identified as Chinese participated in this study. All participants were first-generation immigrants and their average age was 53-years-old. Other demographic characteristics of the sample can be found in Table 1. On the Vancouver Index of Acculturation (VIA; Ryder et al., 2000), the total sample of 13 participants scored higher on heritage culture adherence than mainstream acculturation.

**Table 1:** **Participant demographics**.

Variables	All participants (*N* = 13)	Language spoken in focus group	
		English (*n* = 4)	Mandarin (*n* = 9)
Mean age in years (SD)	52.5(12.9)	37.0(7.8)	59.4(7.2)
Mean years of residency in Canada (SD)	9.7(10.2)	12.0(12.6)	8.7(9.7)
Country of birth			
Taiwan	2	1	1
Hong Kong	2	2	0
China	9	1	8
Acculturation as assessed by VIA			
Mainstream scores (SD)	61.1(17.7)	69.3(8.2)	57.4(19.9)
Heritage scores (SD)	70.4 (11.5)	66.5(7.8)	72.1(12.9)
Marital status^a^			
Never married	2	1	1
Married/common-law/divorced/widowed	9	3	6
Highest educational level attained			
Partial high school	1	0	1
Completed high school	1	0	1
Completed vocational/trade school	3	0	3
Completed undergraduate degree	8	4	4

*^a^Two women did not report their marital status*.

### Measures

#### Vancouver index of acculturation

In accordance with a bidimensional perspective of acculturation, the VIA (Ryder et al., 2000) was administered to separately examine the degree to which participants acculturated to mainstream Canadian culture and adhered to Chinese culture, which is not captured by knowledge of one’s ethnic group. Participants’ acculturation status was especially important to ascertain in this study given that Pap testing behavior was investigated as a cultural phenomenon.

The VIA comprises 20 items, with one heritage and one mainstream item keyed to each of the following 10 domains: cultural traditions, marriage partner, social activities, comfort in professional relationships, entertainment, behavior, maintenance or development of cultural practices, values, humor, and social relationships. Higher scores on the heritage dimension indicated greater adherence to heritage culture and higher scores on the mainstream dimension indicated greater westernization. For both dimensions, scores can range between 0 and 90. Both dimensions of the VIA have been found to have good internal consistency in the Chinese validation sample (Cronbach’s α = .92 for heritage acculturation and .85 for mainstream acculturation).

#### Demographics questionnaire

A questionnaire assessing general demographic characteristics, including age, place of birth, years of residency in Canada, marital status, annual household income, and educational level, was administered.

### Procedure

Participants from a larger, quantitative study (Woo et al., 2012) were given the option to participate in the current study. The quantitative study investigated the relationship between culture, reproductive health behaviors (e.g., Pap testing), and sexuality-related variables. Flyers advertising the quantitative study indicated that research participants were being sought for a study on cancer and culture and that participation entailed an one-on-one interview lasting approximately 60 minutes. In order to participate, participants had to be female, 20-years-old or older, and identify as either Chinese or Caucasian in ethnicity. Women who indicated interest in the current study were contacted by phone and informed of the location and time of the focus groups. Upon arrival, informed consent was obtained from each participant. Participants next took part in a group discussion on Pap testing. Two focus groups were conducted for this study, one in Mandarin (*n* = 9) and one in English (*n* = 4). Women were given the choice to join the Mandarin- or English-speaking group, depending on their language preference and fluency. The first and third authors facilitated the Mandarin-speaking group, with the second author observing and making process notes. The second and third authors facilitated the English-speaking group, with the first author serving the role of silent observer. Both groups were approximately 2 hours. Saturation in themes was reached after just two groups such that the same general content was raised in both groups. The overarching goal of the focus groups was to better understand factors underlying low Pap testing rates among Chinese women. Accordingly, the group facilitators asked participants about the following topics (in order): general understanding regarding Pap testing, personal Pap testing experiences (with focus on their first and last tests), previously encountered internal and external barriers to Pap testing, attitudes toward Pap testing, discussion of Pap testing in their social network (with focus on family, friends, and romantic partner), suggestions on how to increase Pap testing among Chinese women, comparison between Pap testing and other health check-ups and lastly, general health practices, and beliefs. A list of key questions was followed in both groups and appropriate follow-up questions were asked depending on the information shared by participants. After each question was asked, the group facilitators were silent and provided space for participants to share their lived experiences naturally. A natural dialog and interaction among participants were evident in both groups. Following participation, the women were remunerated with $20 and compensated for transportation costs. Both group discussions were audiotaped, transcribed, and translated to enable subsequent data analyses. All procedures were approved by the University’s behavioral research ethics board.

### Data analysis

Thematic content analyses was conducted on the focus group transcripts by four of the authors. These analyses were not restricted to any particular theoretical framework and thus remained largely exploratory. First, the investigators independently read the focus group transcripts without attempting to identify codes or themes. During the second reading, each investigator recorded any significant or interesting impressions in the margins of the text. On the third pass, coders re-read their notes to extract any distinct themes or categories emerging in the focus group discussions. More detailed codes and subcodes were derived during the last reading of the text and notes. Altogether, the investigators compiled a list of 21 possible themes and sub-themes and discussed this list until they agreed on the major themes. Using this agreed-on code, the first author read the focus group transcripts again to code specifically for these themes and identify transcript passages supporting the themes. These passages were recorded on a separate document.

We used two standard methods to establish that intercoder agreement of the qualitative data was reached. First, we used multiple-coding of the same excerpt by the different coders. As a team, we next used a process of discussion to resolve each interpretive discrepancy. A specific score for intercoder agreement was not calculated as the common practice in qualitative analysis is to engage in a dialogic process of interpretive agreement, yielding the highest agreement possible at the end (Marshall and Rossman, 1989). In all cases, the investigators were able to come to agreement.

## Results

Our focus groups comprised Chinese women who were first-generation immigrants and of the average age of 53-years-old. Three major themes emerged from the focus group discussions on Pap testing: preference for Chinese medicine, culturally bound perception of sex and sexuality, and differences in the institutionalization of medical care between country of origin and country of residency.

### Preference for Chinese medicine

One major theme that arose from the focus group discussions was the women’s preference for Chinese medicine over Western medicine. This theme emerged in the women’s stories of their general health practices and beliefs. Aside from explicitly stating their preference for Chinese medicine, the participants also recounted myriad personal experiences to illustrate the superiority of Chinese medicine across a broad range of health topics. Together, the two groups related nine incidences where Chinese medicine was the more effective treatment and none where the opposite was true. One woman noted “in Western medicine, there isn’t any medicine for being tired, but in Chinese medicine, they can prescribe a formula to slowly readjust the balance in your body. It doesn’t work overnight, but it takes care of the root cause” (An, 57 years old, Mandarin-speaking group).

Participants described three main reasons for favoring Chinese medicine. First, to these women, Western medicine seemed to be an enterprise too new to be fully trusted, especially compared to the lengthy history and track record of Chinese medicine. The women also perceived Chinese medicine to be superior over Western medicine because the former has a more holistic conception of health. One participant criticized Western medicine as “to me, it is mind, body, spirit, in one. [The Western medical] approach is to treat things in parts. You get this and that problem, and they would treat that particular problem; it is covering up the symptoms, rather than treating you holistically” (Bik, 37 years old, English-speaking group). Furthermore, to the participants, the emphasis on empiricism severely restricts the scope of Western medicine while Chinese medicine, being open to unseen forces in health, such as *qi* and the balance between *yin* and *yang*, provides a more-rounded and accurate model of health and disease. One experience related by a participant in the Mandarin-speaking group exemplified this:

15 years ago, I started having this lump in the thyroid and it isn’t a cancerous one…. I told the doctor that if the surgery would hurt my voice and ability to sing, I would not do it. However, I did the operation, and after that, I felt that the strength of my voice was weakened…. Western doctors all said that there wasn’t any effect [of surgery], but Chinese doctors say that the strength was obviously weakened. That’s the difference. Western doctors look at things that can be seen and observed; Chinese doctors also look at the things that cannot be seen, like energy (Cuifen, 57 years old, Mandarin speaking group).

When participants were asked how they would guard themselves against developing diseases that may be initially asymptomatic, the influence of Chinese medicine was especially evident as women described taking preventative measures. This focus on prevention instead of screening was in accordance with the orientation of Chinese medicine toward illness prevention, rather than Western approaches to treating already present diseases (Lu et al., 2004). One participant stated:

I think early detection of cancer is difficult, and that’s why I follow a best-selling book titled, “Treat Your Body Rather Than Seek a Doctor”. This book was written by a Chinese traditional medical doctor, and advises on how to keep the body in good shape and prevent illnesses or ailments. The idea is to invest in your health and prevent your body from catching diseases in the first place (Dongmei, 59 years old, Mandarin speaking group).

Other common preventative methods that were mentioned were acupuncture, Chinese herb/food therapy, physical exercise, and maintaining a positive outlook. Interestingly, during these discussions participants continually exchanged Chinese medical health tips, such as eating dates to help strengthen the pancreas and promote blood production. When health tips were shared, they were almost always taken at face value by other participants and regardless of the advice-giver’s lack of medical background. There was very little questioning of how or where these health tips were learned in the first place. Verbal transmission of information such as this may be one mechanism by which the influence of Chinese medicine is maintained in the community.

### Culturally bound perception of sex and sexuality

Unlike the women’s preference for Chinese medicine, which predominantly arose from discussions on general health practices and beliefs, the theme of sex and sexuality emerged in response to discussions of Pap testing. It was evident that participants did not regard the Pap test as just another cancer screening procedure but viewed it through a specifically sexual lens. One woman noted “it [a pap test] is related to any kind of previous experience or any kind of sexually related experience” (Fang, 37 years old, English-speaking group).

The association between Pap testing and sexuality is not surprising given that the Pap test requires exposing one’s genitalia, an area of the body that is normally only revealed to another person during sexual activity, and even then, to a limited degree in some instances. Also, it appeared from the discussions that a link between sexuality and Pap testing exists because sexual promiscuity is believed to be a cause of reproductive tract diseases. For example, one woman described:

In the hospital I was working at, we received one case where a girl about the age of 16, 17, who was educated, came to our hospital with a case of ovarian cyst. It was a bit strange and so everyone was curious about it. But, the doctor told us not to think that she was an indecent girl. You know what I mean by an indecent girl… (Guan-Yin, 52 years old, Mandarin speaking group).

Consistent with traditional Chinese attitudes toward sexuality (Ng and Lau, 1990), participants talked about sexuality in an uneasy, negative, and conservative manner. For example, one participant recalled her experience growing up in China, “I think this barrier is seeded quite deeply because girls and boys were separated in school when we grew up. When I was growing up, my parents would tell me not to play with boys. I grew up in this kind of mentality and habit” (Dongmei, 59 years old, Mandarin-speaking group).

Interestingly, when participants talked about sexual issues, they did not use the word “sex,” and the facilitators interpreted this as avoidance over a concern about being improper. One participant described the traditionalism surrounding discussions of sexuality, “I think Chinese people in Mainland [China] are more conservative and they would not specifically say things; it is part of culture, manners, and habit. A lot of things should not be said” (Guan-Yin, 52 years old, Mandarin-speaking group).

It appeared that because the women associated Pap testing with sexual activity, their discomfort and negativity surrounding sex permeated their view of Pap testing. Like their treatment of the word “sex,” the participants similarly avoided using the term “Pap test” in their discussions. Instead they often referred to it as “the test” or “women’s health check-up.” Again, much like the topic of sexuality, it appeared to be taboo to talk openly about Pap testing. One participant stated “a female worker would usually tell her boss that she is going for a medical exam, and not the specifics. I believe it is Chinese culture not to be too specific and revealing. If you say that you are going for a pap test to your boss, then you probably have to explain what it is for. That is difficult” (Guan-Yin, 52 years old, Mandarin-speaking group).

### Institutionalization of medical care

The third prominent theme that emerged was the differences in the institutionalization of medical care between China and Canada. As such, this theme was only relevant to women who immigrated from China which represented 69.23% of our sample (*n* = 9).

The first difference underscored by the women was that Pap testing and other health screenings are a matter of personal decision in Canada, but are compulsory in China. Many women corroborated this:

In China, we underwent check-ups as told by our “post” (i.e., job position within a company). We had to do these check-ups every year… and the Pap smear was included (Huian, 75 years old, Mandarin-speaking group). If you were within the recommended age range for undergoing the check-ups, they [the company] would assign you to go. So, for example, if you graduated from the university and were in your 20s, then you would likely be assigned for it (Guan-Yin, 52 years old, Mandarin-speaking group).

Furthermore, the women generally expressed preference for the compulsory system in China:

I think the health check-up system here in Canada is not as good as that in China. And this is why my daughter, who is now 40 years old, hasn’t see a doctor in the past years in Canada, except for the time she had her baby… I think the system China has, the compulsory check-up system, is very effective… Maybe they [the Canadian health care system] can have a van that performs pap tests and general check-ups and drive it around to administer these tests (Jun, 59 years old, Mandarin-speaking group).

Having grown accustomed to the compulsory system in China, some women revealed that immigrating to Canada resulted in less frequent health screenings. For example, one woman disclosed “in the past, the company organized it [check-ups] for you. You had a check-up every one or two years. But, I haven’t done one in Canada. I think I am old now and we didn’t discover anything abnormal, so I just leave it” (Dongmei, 59 years old, Mandarin-speaking group).

Another major difference between the health care system in China and Canada that emerged in the group discussions was the environment in which Pap tests are performed. In Canada, Pap tests are usually done in the private office of a primary care provider and there is usually an established relationship between the woman and her physician. In China, Pap tests are conducted at large hospitals amongst a greater battery of tests, and carried out by specialists whom the woman is unlikely to have met. One woman provided an interesting description of what it is like to obtain a Pap test in China, “there are lots of people, and so the procedures at the hospital are the ‘run-of-the-mill’ sort, like a factory” (Kwong, 64 years old, Mandarin-speaking group). Another woman corroborated this description “there were usually many, many people, so patients got pushed along for [Pap] tests very quickly… Everything is about efficiency because there is such a huge population in China!” (Guan-Yin, 52 years old, Mandarin-speaking group).

Again, the women expressed a preference for the Pap testing environment in their home country over the one found in Canada. Women described stories which suggested that the setting in China allowed the women more anonymity as they felt like just another face in the crowd in the large hospital and just a number on the chart to the specialist. This anonymity appeared to diminish the sexual tone of Pap tests, which then buffered the women from feelings of embarrassment and unease. As one participant described:

I think the setting at which the Pap test is done is also a factor that causes embarrassment… In Canada, the family doctor’s clinic is like the size of a small apartment. That’s why I think this setting causes anxieties. I remember in Beijing, we didn’t have such worries! Maybe it was because everything was big and you were just one of the patients among all those who came to see the internal medicine section or the external medicine section. Here, the rooms are tiny, and so you would have these thoughts. In China, because there were so many doctors, you would rarely run into the same one. Plus, the doctor had a lot of patients, so it was not unusual that patients come and go. So, that’s why I think this environment didn’t impose stress and anxiety on you. In Canada, you go back to the same family doctor time after time (Guan-Yin, 52 years old, Mandarin-speaking group).

## Discussion

Research on the low Pap testing rate among Chinese women has been significantly underserved by qualitative approaches. The aim of the current study was to bridge knowledge gaps in this research area by gathering qualitative data and improving our understanding of the lived experiences of Chinese women in relation to Pap testing. In this study, thematic content analyses were performed on transcripts of focus group discussions on Pap testing and other health practices. The focus groups consisted of first-generation Chinese immigrant women, who were of the average age of 53 years old. Our analyses revealed three major themes in the women’s discourse: influence of Chinese medicine, culturally bound perceptions of sex, and differences in the institutionalization of medical care.

### Influence of Chinese medicine

With respect to the first theme, the women expressed a strong preference for Chinese medicine over Western medicine and delineated three main reasons for their bias: Chinese medicine has a longer history, allows for influences on health that may be neither visible nor detectable with available technologies, and views health more holistically. Moreover, when asked about how they would protect against developing diseases that are asymptomatic, participants primarily focused on holistic preventive measures such as eating certain foods or herbs, which dovetails with the ideology of Chinese medicine.

Our findings support similar findings by others (Jackson et al., 2002); however the current study differs regarding the specific nature of the influence of Chinese medicine. Whereas we did not find belief in Chinese medicine to be a theme in the discourse on Pap testing but only when the topic was broadened to general health, Jackson et al. (2002) observed Chinese medicine to be a central theme in the women’s narratives on Pap testing. Specifically, they found that specific ideas of Chinese medicine, such as *qi* circulation and bodily balance, directly countered the logic of Pap testing. Our study, however, suggests that the effect of Chinese medicine is broader, affecting a wide range of health domains, of which cervical health is just one. Preference for Chinese medicine may deter Chinese women from following health advice from Western doctors, including recommendations to obtain Pap tests. The orientation of Chinese medicine toward holistic preventive measures may also lead Chinese women to engage in preventative measures, such as eating healthful foods, to protect against cervical cancer instead of regular Pap tests. Based on the women’s reasons for preferring Chinese medicine, we speculate that an additional cause of Chinese women’s low Pap testing behavior is its non-holistic nature and focus on visible signs of disease (i.e., detecting signs of an already present disease in a specific body region).

### Culturally bound perception of sex and sexuality

In addition to finding support for the negative influence of Chinese medicine on Pap testing, our study also identified two novel deterrents: culturally bound perceptions of sex and sexuality and differences in the infrastructure of health care.

We found that Chinese women make a close association between Pap testing and sexuality. One basis for this association appears to be their belief that reproductive diseases are caused by sexual promiscuity. Through this connection between sexuality and Pap testing, Chinese women’s culturally bound negative views of sexuality seem to permeate their attitudes toward Pap testing. There were striking similarities in how Chinese women talked about sexuality and Pap testing. Both subjects were discussed with minimal use of the terms “sexuality” and “Pap testing.” Participants also considered both sexuality and Pap testing to be too taboo for frank discussions. Such treatment of sexuality and Pap testing exemplifies the “high-context” nature of Chinese culture. In contrast to “low-context” Western culture where topics are directly approached, Chinese individuals are expected to communicate in indirect, implicit, and non-verbal ways and in turn, listeners are expected to attend to these cues and discern their meanings (Kim and Ward, 2007). Through this style of communication, sociocultural messages about sexuality, Pap testing, and other taboo topics can be imparted without explicit statement.

Previous research has revealed that embarrassment is more likely to discourage Chinese women than Euro-Canadian women from seeking a Pap test (Woo et al., 2009). Based on the current study, we postulate that one source of embarrassment for Chinese women stems from their culturally bounded perception of sex and sexualization of Pap testing. To date, only one study has investigated the role of sexuality in Pap testing and found that neither sexual function nor knowledge predicted Pap testing behavior (Woo et al., 2009). We believe that future research examining the relationship between Pap testing and sexuality in Chinese women may be more fruitful if it examines the influence of the sexualization of Pap testing and sexual traditionalism on Pap testing behavior. Although ancient Chinese society was relatively sexually open (e.g., the world’s oldest sex manuals are Chinese; Ruan, 1991), neo-Confucian scholars gave Confucian teachings strict interpretations in the Song Dynasty which have maintained their stronghold in Chinese culture, particularly regarding the reservation of sexual activity for marriage (Ng and Lau, 1990). This sexually suppressive social environment is likely to deter unmarried Chinese women from undergoing Pap tests.

### Differences in the institutionalization of health care

Another prominent theme that emerged from the women’s discourse centered on disparities between the Canadian and Chinese health care infrastructures. In China, women underwent Pap testing in a compulsory (i.e., required and organized by their employer) and anonymous (i.e., conducted in a large hospital by an unknown specialist) manner. Importantly, the women primarily preferred this method of health care administration. Some women further added that immigrating to Canada has been accompanied by a decrease in regular screenings. The women’s preference may come as a surprise since according to previous research, patient satisfaction, and compliance are higher when they perceive that their wishes are heard and their rapport with their physician is strong (Jin et al., 2008). However, the majority of this research was conducted with patients of WESTERN backgrounds and the generalizability of results to other cultures is unknown. The present study would suggest that these results are not generalizable to Chinese women in the context of Pap testing. Drawing on the current findings as well as the findings of prior research (e.g., Woo et al., 2009), Chinese women may favor compulsory and anonymous Pap testing as these features diminish the sexually charged meaning of and embarrassment surrounding Pap testing. With respect to freedom of choice in undergoing testing, compulsory Pap testing renders a woman’s sexual history irrelevant; instead of the Pap test being a test that a woman chooses to undergo because she is sexually active, it becomes a test that she is undergoing because she is told to do so. Much like the anonymity that is associated with HIV testing (Myers et al., 1993; Bindman et al., 1998), anonymity with Pap testing may also lessen the embarrassment attached to getting a Pap test and thus encourage its usage among Chinese women.

In summary, this study has identified three aspects of Chinese culture that may help explain the phenomenon of lower Pap testing rates among middle-aged, first-generation Chinese immigrant women. First, the influence of Chinese medicine philosophy may lead these Chinese women to question the utility of Pap testing and perceive it as a Western medical practice flawed in its contradiction to the holistic, prevention-centric Chinese model of health. Chinese women’s motivation to obtain a Pap test may be further inhibited by the link they draw between Pap testing and sexuality, the latter of which is often viewed negatively by their culture. This problematic association is further exacerbated by the individualized and voluntary manner in which Pap testing is administered by the Western health care institution. In contrast, the anonymous and compulsory nature of Pap testing in China appears to buffer Chinese women from the embarrassment and sexually charged meaning of Pap testing. From the women’s discourse, there overall appears to be a cyclical relationship between health practices and cultural values in that they are a reflection and product of each other. Findings from this study generally echo what has been found with other cancer screening practices in Chinese patients. With respect to the influence of Chinese medicine, past research has also found it to discourage, rather than promote, cancer-screening behavior. This has been attributed to the incompatibility between the concept of disease detection and philosophies of Chinese medicine (Liang et al., 2004). Not surprisingly, previous studies suggest that embarrassment also plays a role in the cancer screening of other commonly sexualized body areas. For example, among Chinese women, embarrassment has consistently been found to be a deterrent of undergoing mammograms (Mo, 1992) and colorectal cancer screening (Hou, 2005). To our knowledge, differences in the institutionalization of health care have not been previously identified as a barrier to cancer screening and our findings suggest that this may be studied as a potential moderator of behavior.

### Limitations

The results of the current study should be interpreted with the following limitations in mind. First, only two focus groups consisting of 13 women took place. This concern of small sample size may be mitigated by the fact that saturation in themes was reached after the two focus groups. Also, the inevitable trade-off between the number of focus groups and the depth of description also contributed to our decision to stop recruitment after theme saturation at two groups as the quality of qualitative studies significantly depends on the richness of the data and its analysis (Carlsen and Glenton, 2011).

In addition, the women in the focus groups were mostly from China, middle-aged, married, or had been married and were all first-generation immigrants. Thus, our results may not generalize to and adequately inform Pap testing initiatives for Chinese women who are from Asian countries outside of China, young, never married, or born in Canada. Furthermore, we did not gather information on which cities the participants were from, beyond their countries of origin. As a result, our study cannot reveal potential within-country geographic variations regarding Pap testing attitudes and experiences, such as those between urban and rural areas. Our study also did not utilize random sampling from the population but instead relied on volunteers who were willing to discuss issues relating to sexuality with strangers. This introduces volunteer bias, which also endangers the generalizability of our results.

### Implications

Despite its limitations, the current study identified three important culture-specific impediments to Pap testing among Chinese women: influence of Chinese medicine, culturally bound perception of sex and sexuality, and differences in the institutionalization of medical care. Future interventions targeted toward raising Pap testing among Chinese women of similar demographics as our participants (e.g., first-generation immigrants, middle-aged) would likely be more effective if they were culturally sensitive, while at the same time, addressing these three barriers. First, efforts could be made to reconcile the importance of Pap testing with philosophies of Chinese medicine. Based on the current study, Chinese women seem to pit Western and Chinese medicine against each other and view them as antithetic. If intervention programs can encourage Chinese women to view Western medicine as complementary to Chinese medicine, they may become more open to Western health concepts and practices, including Pap testing. For example, Chinese women could be encouraged by health professionals to adopt regular Pap testing into their health regimen as part of a larger program of holistic health practices, including Chinese preventative methods. Moreover, previous research has demonstrated that health educational programs are more effective when the audience perceives the educator as a well-informed expert (Hilton, 2003; Strange et al., 2003). While physicians and nurses are usually effective in this role, Pap testing programs aimed at Chinese women may consider including Chinese medicine practitioners as educators since Chinese women may perceive them as more knowledgeable than Western physicians. Chinese medicine practitioners may also be perceived as possessing a better understanding of health on a holistic basis. In addition, given our observation of the effectiveness of verbal exchange of health tips among group participants, efforts aimed at increasing Pap testing among Chinese women may be more effective using this mode of communication. For example, the adoption of a more informal, conversational manner to discuss health benefits of Pap testing may be more persuasive than the lecture style typical of workshops and programs.

Second, endeavors directed toward promoting Pap testing among Chinese women could address the perceived sexualization of Pap testing. One way to do so is by educating Chinese women about the etiology of gynecological disease and dispelling the erroneous belief that sexual promiscuity is a cause. Furthermore, physicians could be made aware of the sexual meaning of Pap testing to Chinese women. Specifically, physicians need to understand the difficulty of talking about Pap testing for Chinese women and thus proactively broach the topic with their patients. During these conversations, physicians could talk about Pap testing in a matter-of-fact fashion. Any signs of unease or embarrassment by the physician may confirm a woman’s pre-existing trepidation about the sexual undertones of this behavior.

In light of what this study found about China’s health care infrastructure, another promising way of increasing Pap testing among Chinese women may involve changing the way that Pap testing is conducted. Specifically, Chinese women may be more likely to regularly undergo Pap tests if they had the option to do so in a more anonymous setting, such as in a large hospital by an unfamiliar physician. Moreover, having undergone compulsory screening in their country of origin, Chinese women may have become accustomed to taking a passive role in the matter of Pap testing. Given their preference for compulsory screening, programs designed to empower Chinese women to take charge of their own cervical health may not be where we want to focus our intervention endeavors. Instead, our efforts may be better directed at informing physicians to take a more directive approach when encouraging Chinese women to obtain Pap tests as such persuasion from authority figures may mimic the effect of compulsory testing.

### Future directions

Future research should focus on translating these qualitative findings into testable, quantitative hypotheses. Moreover, there is an important knowledge-to-action aspect of the findings that may inform the development of more effective interventions to improve Pap testing rates. Also, follow-up qualitative research can help determine the replicability of the three themes found in this study. Such studies, by using larger, more homogenous (e.g., participants from the same country or city) samples, would be able to draw stronger conclusions. Comparisons between these studies would allow a better understanding of geographic variations in attitudes toward Pap testing within countries and between urban and rural regions. The richness of themes gleaned from this study should also encourage future qualitative research in Pap testing and sexuality in different groups of ethnic minority women.

## Conflict of Interest Statement

The authors declare that the research was conducted in the absence of any commercial or financial relationships that could be construed as a potential conflict of interest.
